# 3D-printed stretchable modular integrated microsystems toward sweat monitoring powered by wireless charging sodium-ion micro-batteries

**DOI:** 10.1093/nsr/nwaf364

**Published:** 2025-08-30

**Authors:** Zhihao Ren, Xiaoyu Shi, Endian Yang, Lanxiu Ni, Zhuobin Guo, Bin Li, Yin Wu, Yuxin Ma, Junwei Sun, Yangyang Liu, Chunsheng Li, Jiaxin Ma, Xiao Wang, Feng Zhou, Fangyuan Hu, Liang Feng, Quan Shi, Zhong-Shuai Wu

**Affiliations:** State Key Laboratory of Catalysis, Dalian Institute of Chemical Physics, Chinese Academy of Sciences, Dalian 116023, China; University of Chinese Academy of Sciences, Beijing 100049, China; State Key Laboratory of Catalysis, Dalian Institute of Chemical Physics, Chinese Academy of Sciences, Dalian 116023, China; Dalian National Laboratory for Clean Energy, Chinese Academy of Sciences, Dalian 116023, China; State Key Laboratory of Catalysis, Dalian Institute of Chemical Physics, Chinese Academy of Sciences, Dalian 116023, China; Department of Instrumentation and Analytical Chemistry, CAS Key Laboratory of Separation Science for Analytical Chemistry, Dalian Institute of Chemical Physics, Chinese Academy of Sciences, Dalian 116023, China; State Key Laboratory of Catalysis, Dalian Institute of Chemical Physics, Chinese Academy of Sciences, Dalian 116023, China; University of Chinese Academy of Sciences, Beijing 100049, China; State Key Laboratory of Catalysis, Dalian Institute of Chemical Physics, Chinese Academy of Sciences, Dalian 116023, China; University of Chinese Academy of Sciences, Beijing 100049, China; State Key Laboratory of Catalysis, Dalian Institute of Chemical Physics, Chinese Academy of Sciences, Dalian 116023, China; State Key Laboratory of Catalysis, Dalian Institute of Chemical Physics, Chinese Academy of Sciences, Dalian 116023, China; University of Chinese Academy of Sciences, Beijing 100049, China; State Key Laboratory of Catalysis, Dalian Institute of Chemical Physics, Chinese Academy of Sciences, Dalian 116023, China; State Key Laboratory of Catalysis, Dalian Institute of Chemical Physics, Chinese Academy of Sciences, Dalian 116023, China; Department of Instrumentation and Analytical Chemistry, CAS Key Laboratory of Separation Science for Analytical Chemistry, Dalian Institute of Chemical Physics, Chinese Academy of Sciences, Dalian 116023, China; State Key Laboratory of Catalysis, Dalian Institute of Chemical Physics, Chinese Academy of Sciences, Dalian 116023, China; Dalian National Laboratory for Clean Energy, Chinese Academy of Sciences, Dalian 116023, China; State Key Laboratory of Catalysis, Dalian Institute of Chemical Physics, Chinese Academy of Sciences, Dalian 116023, China; Dalian National Laboratory for Clean Energy, Chinese Academy of Sciences, Dalian 116023, China; State Key Laboratory of Catalysis, Dalian Institute of Chemical Physics, Chinese Academy of Sciences, Dalian 116023, China; Dalian National Laboratory for Clean Energy, Chinese Academy of Sciences, Dalian 116023, China; School of Materials Science and Engineering, State Key Laboratory of Fine Chemicals, Dalian University of Technology, Dalian 116024, China; Department of Instrumentation and Analytical Chemistry, CAS Key Laboratory of Separation Science for Analytical Chemistry, Dalian Institute of Chemical Physics, Chinese Academy of Sciences, Dalian 116023, China; Thermochemistry Laboratory, Dalian Institute of Chemical Physics, Chinese Academy of Sciences, Dalian 116023, China; State Key Laboratory of Catalysis, Dalian Institute of Chemical Physics, Chinese Academy of Sciences, Dalian 116023, China; Dalian National Laboratory for Clean Energy, Chinese Academy of Sciences, Dalian 116023, China

**Keywords:** stretchable electronics, 3D printing, wireless charging, sodium-ion micro-battery, glucose sensor

## Abstract

The emerging development of energy storage integrated microsystems plays a pivotal role in advancing flexible and wearable electronics, yet their compatible construction and stretching robustness remain unsolved. Herein, we report an all three-dimensional (3D) printing construction of a stretchable modular integrated microsystem, composed of wireless receiving coils, aqueous sodium-ion micro-batteries (ASMBs) and glucose sensors. A versatile binder-free graphene-based ink is developed to largely reduce the construction complexity and boost compatibility between multiple modules. The resulting 3D-printed ASMBs exhibit high areal capacity of 0.96 mAh cm^−2^, favorable stability after 1500 cycles and uniformity of integration, profited from the elaborately designed microelectrodes with fast electron/ion transfer pathways. By charging in contactless mode, our ASMBs can efficiently drive a sensor to realize highly sensitive detection of glucose concentrations as low as 0.5 mM. When encapsulated within an Ecoflex elastomer and connected with liquid metal, the microsystems maintain invariable operation after thousands of stretching deformations at 50% tensile strain. Our proposed strategy of all 3D-printing microfabrication and structure design paves a way for developing highly flexible and customized integrated microsystems toward wearable electronics.

## Teaser

A highly compatible and stretchable modular integrated microsystem, consisting of wireless receiving coils, aqueous sodium-ion micro-batteries, and glucose sensors, is constructed through 3D printing of versatile binder-free graphene-based inks.

## INTRODUCTION

With the continuous improvement of living standards, people's awareness of personal health has gradually increased. This has driven the rapid development of flexible and wearable electronic devices to monitor a wide range of physiological indicators of the human body in real-time by means of various sensors, then supporting precise and personalized treatments, and also complementing traditional hospital diagnosis [1–5]. Meanwhile, this trend has posed significant challenges for energy storage devices. Conventional electrochemical energy storage devices (e.g. coin cells, pouch cells) usually exhibit large volume, heavy weight and rigid structure, making them difficult to meet the application requirements of wearable electronics [6,7]. To address this issue, planar microscale electrochemical energy storage devices (MEESDs), including micro-batteries (MBs) and micro-supercapacitors, have garnered extensive attention in recent years [8–11]. Their unique structure characteristics, with positive and negative electrodes positioned on the same plane, free of separators, could effectively mitigate interface separation during deformation and facilitate smooth integration with other components, which has opened up new opportunities for the development of flexible and wearable electronics. Furthermore, with the progress in terms of electrochemical energy storage systems, high-performance electrode and electrolyte materials, and microfabrication techniques, the performance of MEESDs has been steadily improved [12–15].

Moreover, the function and integration characteristics of MEESDs have also been significantly boosted. On one hand, energy harvesting devices, such as solar cells, nanogenerators and wireless charging modules, have been introduced to address the incompatibility issue between MEESDs and traditional charging schemes [16–18]. They can convert other forms of energy into electrical energy to charge MEESDs to compensate for their limited capacity, while avoiding cumbersome contact charging modes. On the other hand, the functions of MEESDs have also been greatly expanded, through integration with various sensing materials and devices (e.g. body temperature, sweat and movement sensors) [19–22]. Furthermore, self-powered integrated microsystems consisting of energy harvest, energy storage and energy consumption units have been preliminarily demonstrated, which has greatly accelerated the progress of MEESDs for flexible electronics [23–25].

Despite significant advancements, several key issues currently remain unsolved. First, the multiple modules in the integrated microsystems are usually constructed by different technical solutions, resulting in poor compatibility between these components and considerable inconvenience [26,27]. Second, most integrated microsystems lack efficient stretchability, making them unsuitable for skin-attachable wearable applications [27,28]. Therefore, there is an urgent need to develop a feasible construction solution for stretchable integrated microsystems that is fully compatible in terms of key materials, multiple interfaces between different modules, and microfabrication techniques.

In this work, we developed a highly compatible and stretchable modular integrated microsystem through all three-dimensional (3D) printing of versatile binder-free graphene-based inks. Specifically, the 3D-printed aqueous sodium-ion MBs (ASMBs) exhibited high areal capacity (0.96 mAh cm^−2^), favorable cyclability (1500 cycles), integrated uniformity and mechanical robustness after 1000 times of stretching at 50% tensile strain. Simultaneously, the 3D-printed graphene patterns served as wireless receiving coils and the carrier of glucose sensing materials, greatly reducing the construction complexity of the microsystem and enhancing compatibility between different modules. Furthermore, after connecting the above components via liquid metal, our ASMBs were successfully charged by the wireless receiving coils and sequentially powered the NiCo_2_O_4_ (NCO) sensor to enable highly sensitive and rapid detection of glucose. Moreover, the modular integrated microsystem maintained an almost unchanged performance after repeated stretching deformations, demonstrating the application potential in real-world scenarios. Therefore, this work presents a 3D-printing paradigm to construct customizable and highly stretchable integrated microsystems.

## RESULTS AND DISCUSSION

### All 3D-printing construction of the stretchable microsystem

**Figure 1.**
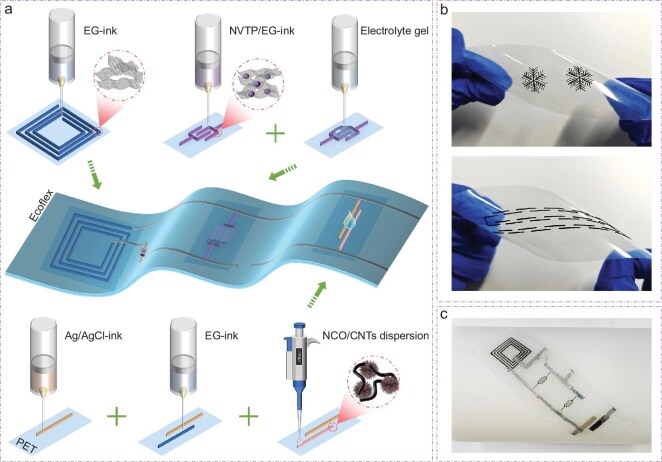
Fabrication process of the stretchable modular integrated microsystem and 3D-printed patterns. (a) Schematic illustration of the fabrication process for the stretchable microsystem. (b, c) Photographs of the 3D-printed patterns (b) and the stretchable microsystem (c) in a bent state.

Figure 1a shows the fabrication scheme of the stretchable modular integrated microsystem, mainly consisting of the energy harvest module (wireless receiving coils), the energy storage module (ASMBs) and the energy consumption module (NCO glucose sensors), all of which were constructed through 3D-printing. First, we prepared electrochemically exfoliated graphene (EG) in a NaBF_4_ aqueous solution. Then, a low-cost and environmentally friendly glycerol with suitable surface energy was selected as a dispersant to prepare a binder-free EG-based ink (EG-ink) [29]. Based on this, various patterns including the snowflakes, linear shapes, and ‘DICP’ letters were 3D-printed directly onto a polyethylene terephthalate (PET) substrate (Fig. 1b and Fig. S1), indicating excellent processability of the EG-ink and favorable customizability of the 3D-printed patterns. Importantly, no fracture was observed on the printed patterns under twisting deformation, suggesting that this strategy can be used to prepare flexible devices to a certain extent. It is noted that the binder-free feature could effectively improve conductivity, so the 3D-printed EG patterns were directly used as the wireless receiving coils, which is conducive to improving the energy conversion efficiency of the wireless charging process. Further, the carbon coated Na_2_VTi(PO_4_)_3_ (NVTP@C) was prepared to form a composite ink with EG in glycerol (NVTP/EG-ink), for the construction of the microelectrodes. Then, the ASMBs were obtained after 3D-printing of the gel electrolyte onto the microelectrode region. With regard to the glucose sensor module, the working electrode was prepared by drop-casting the NCO/carbon nanotubes (CNTs) hybrid materials onto the 3D-printed EG carrier, and an Ag/AgCl reference electrode was 3D-printed next to the working electrode to enable the sensing function. Finally, the three 3D-printed modules, connected with liquid metal, were encapsulated together using highly elastic Ecoflex films, thus completing the construction of a stretchable modular integrated microsystem (Fig. 1c).

### Rheological properties and printability of EG-ink and NVTP/EG-ink

**Figure 2.**
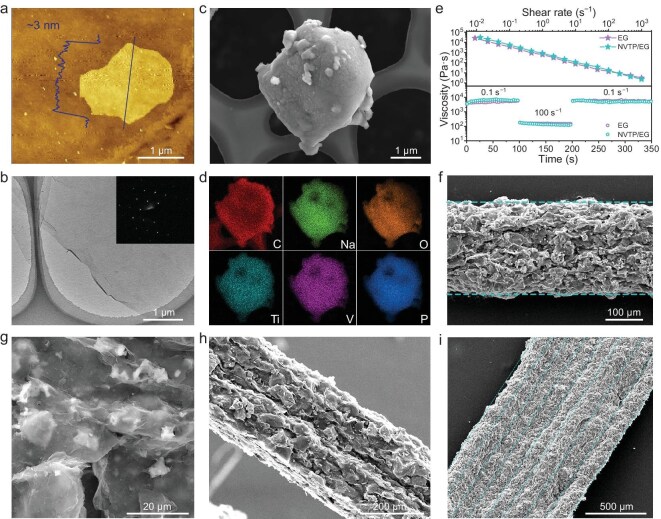
Characterization of electrode materials and printability of inks. (a) Atomic force microscope (AFM) image of EG nanosheets with the corresponding height profile (inset). (b) Transmission electron microscope (TEM) image of EG nanosheets and the selected area electron diffraction pattern (inset). (c) Scanning electron microscope (SEM) image of the NVTP@C electrode material and (d) corresponding energy dispersive spectroscopy mapping. (e) Rheological behavior of EG-ink and NVTP/EG-ink. (f) Top-view SEM image of the 1-layer NVTP/EG electrode. (g) SEM image showing EG and NVTP on the surface of the NVTP/EG electrode. (h, i) Side-view SEM images of NVTP/EG electrodes with 2 layers (h) and 8 layers (i).

The obtained EG displayed a lateral size of 2–3 μm and a thickness of ∼3 nm (Fig. 2a, b), and a diffraction peak at 26.4° in X-ray diffraction (XRD) patterns, as well as the typical D and G peaks in the Raman data (Fig. S2) [30]. These characterization results showed the high quality of the obtained EG nanosheets, thereby ensuring the smooth printing process and high conductivity of the 3D-printed electrodes (Fig. S3). For the energy storage units, we prepared NASICON-type NVTP@C as the active electrode materials. This is because NVTP@C materials possessing multiple redox pairs with large voltage difference can be used as the positive and negative electrodes simultaneously, thereby reducing the difficulty of ink preparation, simplifying the 3D-printing process, and ensuring the uniform performance of MBs. The XRD results (Fig. S4) confirmed the synthesis of NVTP@C, with a particle size ∼2 μm, and energy dispersive spectroscopy mapping suggested uniformly distributed C, Na, Ti, V, O and P elements (Fig. 2c, d). It is worth noting that the NVTP@C displayed a high voltage plateau at 3.3 V, along with two low voltage plateaus at 2.2 V and 1.6 V (Figs S5, S6), corresponding to the V^4+^/V^3+^, as well as Ti^4+^/Ti^3+^ and V^3+^/V^2+^ redox couples, respectively, which lays the foundation for constructing symmetric ASMBs. Moreover, the minimal capacity loss and polarization of NVTP@C under high current densities in different voltage regions further guarantee the feasibility of constructing high-power ASMBs.

To evaluate their suitability for 3D-printing, we carried out the rheological characterization of the binder-free EG-ink and NVTP/EG-ink (Fig. 2e). It is observed that both inks exhibited obvious shear-thinning behavior as the shear rate increased, which facilitates their smooth extrusion during the printing process. Moreover, when switching the shear rate, the viscosity of the two inks gave out a rapid response, allowing for quick solidification of printed patterns on the substrate, and effectively preventing structure collapse. Therefore, the EG and NVTP/EG electrodes displayed a high 3D-printing resolution of 160 μm and 175 μm (Fig. S7), respectively. In our ASMBs, the NVTP/EG electrodes possessed a width of ∼250 μm with a flat and uniform surface (Fig. 2f and Fig. S8). Besides, it can be observed from the side-view (Fig. 2g) and cross-section (Fig. S9) scanning electron microscope (SEM) images that EG nanosheets interlaced each other and NVTP@C particles were tightly wrapped by these EG nanosheets, which is beneficial to forming highly electronic/ionic conductive pathways, that make full utilization of the capacity of active materials. Moreover, from the results of multi-layer printing, it is manifested that the thickness and loading of the microelectrodes displayed an approximately linear increase with the printed layer number, and adjacent layers were closely connected (Fig. 2h, i and Fig. S10). This is conducive to constructing high areal capacity ASMBs with thick electrodes via repeated multi-layer 3D-printing.

### Electrochemical properties of the ASMBs

**Figure 3.**
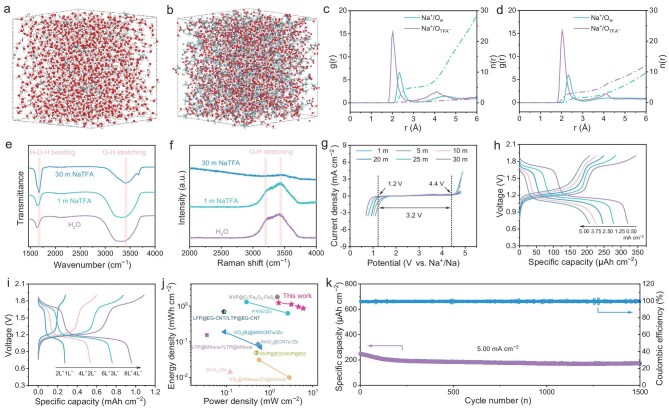
Electrochemical performance of the NVTP/EG ASMBs. (a, b) Solvation structures of 1 m (a) and 30 m (b) NaTFA electrolytes. White, red, purple, blue and grey spheres represent H, O, Na, F and C atoms, respectively. (c, d) Radial distribution functions and coordination numbers of 1 m (c) and 30 m (d) NaTFA electrolytes. (e) FTIR and (f) Raman spectra of H_2_O, 1 m, and 30 m NaTFA electrolytes. (g) LSV curves of NaTFA electrolytes with different concentrations. (h) Rate capability of the ASMBs-2L^+^1L^−^. (i) Specific capacity-voltage curves of ASMBs with varying layer numbers. (j) Ragone plot of ASMBs-8L^+^4L^−^ compared with recently reported MBs. (k) Cyclic stability of the ASMBs-2L^+^1L^−^.

An environmentally friendly and non-toxic aqueous high-concentration electrolyte (30 m sodium trifluoroacetate, NaTFA) was designed for the ASMBs, considering the stringent safety requirements in wearable applications and high operation voltage of the symmetric devices [31–33]. To illustrate the interactions between different components, molecular dynamics simulations were conducted to analyze the solvation structures of 1 m and 30 m NaTFA electrolytes (Fig. 3a, b). The results showed that in 1 m NaTFA (Fig. 3c), the first solvation shell of Na^+^ contained many water molecules (coordination number of ${{\mathrm{O}}}_{\mathrm{w}}$ was 5.1) and less anions (coordination number of ${{\mathrm{O}}}_{{\mathrm{TF}}{{\mathrm{A}}}^ - }$was 0.3), indicating that there existed a large amount of free water. In contrast, the first solvation shell of Na^+^ in the 30 m NaTFA contained 1.7 ${{\mathrm{O}}}_{\mathrm{w}}$ and 3.4 ${{\mathrm{O}}}_{{\mathrm{TF}}{{\mathrm{A}}}^ - }$ (Fig. 3d). It is suggested that the amount of free water was drastically reduced in the high-concentration electrolyte, which is helpful in widening the electrochemical stability window. From the Fourier transform infrared (FTIR) spectra (Fig. 3e), it is revealed that the characteristic O-H stretching vibrations (3200–3400 cm^−1^) and H-O-H bending vibrations (1600–1700 cm^−1^) of H_2_O were noticeably shifted to higher wavenumbers, indicating enhanced covalent bonding within water molecules and improved electrochemical stability [34]. Additionally, as the concentration increased from 1 m to 30 m, the two broad Raman peaks located at 3200 cm^−1^ and 3400 cm^−1^, corresponding to O-H stretching vibrations, gradually transformed into narrower and less distinct peaks accompanied by a blue shift (Fig. 3f), signifying a reduction in the proportion of free water molecules [35]. Linear sweep voltammetry (LSV) measurements further confirmed the aforementioned results. It is observed that compared to other concentrations of electrolytes, the electrochemical stability window of 30 m NaTFA was expanded to 3.2 V (Fig. 3g), fully covering the three voltage plateaus of the NVTP@C. This indicates a perfect match between the electrode and electrolyte, ensuring stable operation of the ASMBs within the targeted voltage range and maximizing the electrochemical performance of the NVTP@C electrode in the aqueous system. Moreover, considering the low cost of NaTFA and its accepted ionic conductivity at high concentrations (Fig. S11), a 30 m NaTFA electrolyte was selected for this study. To prevent the flow and leakage of electrolyte, an appropriate amount of polyvinyl alcohol (PVA) was introduced to endow the electrolyte with favorable rheological properties, making it suitable for 3D-printing (Fig. S12).

It should be pointed out that, considering the capacity difference of the NVTP@C as positive and negative electrodes (Figs S5, S6), capacity matching was achieved by 3D-printing two layers of positive electrode corresponding to one layer of negative electrode (denoted as ASMBs-2*x*L^+^*x*L^−^, where *x* represents the layer number of the negative electrode). As shown in Fig. 3h, the ASMBs-2L^+^1L^−^ exhibited two stable voltage plateaus at ∼1.1 V and ∼1.7 V, consistent with the results in half-cell tests. This is also proven by the cyclic voltammetry (CV) curves in Fig. S13. In addition, the ASMBs-2L^+^1L^−^ gave out a high specific capacity of 320.3 μAh cm^−2^ (14.8 mAh cm^−3^) at a current density of 0.5 mA cm^−2^, and maintained a capacity of 206.9 μAh cm^−2^ (9.6 mAh cm^−3^) even at a current density of 5.0 mA cm^−2^ (∼20 C), indicative of excellent rate capability (Fig. S14). This result also correlated with its relatively low electrochemical impedance (Fig. S15). Furthermore, we tried to achieve capacity customization by varying the 3D-printing layer number. From Fig. 3i, it can be seen that the areal capacity of ASMBs delivered an approximately linear increase as the layer number increased, reaching a maximum discharge capacity of 0.96 mAh cm^−2^ (12.2 mAh cm^−3^) for the ASMBs-8L^+^4L^−^. It is worth noting that the non-ideal linear growth and the slight decline in volumetric performance are attributed to the incomplete utilization of the active material as the electrode thickness increases. Nevertheless, the ASMBs-8L^+^4L^−^ also expressed outstanding rate performance, showing an energy density of 1.24 mWh cm^−2^ at a power density of 1.62 mW cm^−2^, and delivering 0.87 mWh cm^−2^ at a high power density of 6.01 mW cm^−2^, outperforming most previously reported MBs (Fig. 3j) [26,36–43]. In addition, excellent cyclability with 70% capacity retention after 1500 cycles was also demonstrated in our ASMBs (Fig. 3k), implying its reliability in long-term operation. Impressively, the ASMBs achieved a capacity retention of 98.8% after 500 cycles at −15°C and a high capacity of 178.9 μAh cm^−2^ even at −20°C (Fig. S16), significantly broadening the potential application scenarios of the device. These exceptional performances are attributed to rational design and perfect matching of the 3D-printed microelectrodes and the aqueous electrolyte.

### Integration and stretchability of the ASMBs

**Figure 4.**
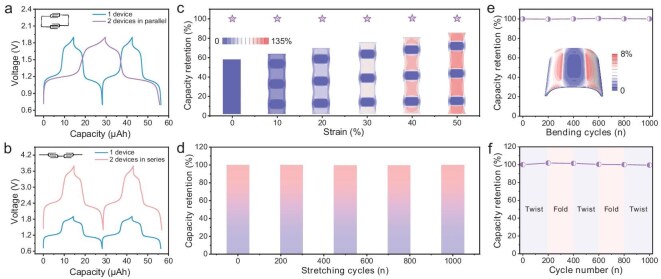
Integration and flexibility of the NVTP/EG ASMBs. (a, b) Galvanostatic charge-discharge profiles of two ASMBs connected in parallel (a) and in series (b), in comparison to single device. The insets are corresponding photographs. (c) Capacity retention of stretchable ASMB arrays connected in parallel at various stretching strains. The insets show corresponding FEA results. (d) Capacity retention of stretchable ASMB arrays connected in parallel after different cycles of 0%–50% stretching. (e) Capacity retention of a single ASMB encapsulated by elastomer after different bending cycles. The insets show corresponding FEA results. (f) Capacity retention of an ASMB after varying cycles of alternating twisting/folding deformations.

In addition to electrochemical properties, the integration features and flexibility of MEESDs significantly influence their application as micropower sources for wearable electronics. In this work, the integrated array of modules by connecting multiple ASMBs in series and in paralell could be easily achieved by adjusting the printing path in the digital 3D-printing strategy. As shown in Fig. 4a, the two ASMBs in parallel connection represented an unchanged voltage platform and doubled discharge capacity compared to the single cell. Relatively, the integrated module composed of serially connected two cells showed a twofold voltage platform whilst keeping consistent charge-discharge capacity (Fig. 4b), which is suggestive of excellent performance uniformity of 3D-printed ASMBs.

Next, we employed Ecoflex elastomer as the encapsulation material to construct a device array with three parallelly-connected ASMBs by virtue of a liquid metal connection. Impressively, the integrated ASMBs exhibited no attenuation in electrochemical performance within the stretching strain range from 10% to 50% (Fig. 4c). More importantly, even after 1000 stretching cycles at 50% tensile strain, the module device still showed consistent charge/discharge behavior with nearly unchanged capacity (Fig. 4d), highlighting the superior mechanical stability of the integrated ASMBs. Similarly, the array device with three ASMBs connected in series also maintained stable performance under the same tests (Fig. S17), which was the first demonstration of such a flexible MEESD. It should be pointed out that the 50% tensile strain at the system level is sufficient to meet the requirements of skin-attachable flexible electronics, because the typical tensile deformation of human skin is generally <30% [27]. Besides, it is revealed that the ASMBs displayed ∼100% capacity retention under 180° bending deformation (Fig. 4e) as well as alternate twisting/folding deformations (Fig. 4f), further proving the applicability of our flexible MEESDs in wearable electronics. Such excellent mechanical robustness is mainly derived from an ‘island-bridge’ structure design that embeds the rigid components into a flexible connector, in which the ASMBs act as the ‘island’ to provide capacity, and the elastomer and liquid metal act as the ‘bridge’ to accommodate strain, thereby endowing superior stretchability in the integrated system [44,45]. From the results of finite element analysis (FEA) at stretching (Fig. 4c, inset) and bending (Fig. 4e, inset) states, the strain was predominantly localized in the elastomer connecting the ASMBs, while the PET remained virtually unaffected. Under the stretching state of 50% tensile strain, the maximum strain (∼135%) on the Ecoflex elastomer was detected at the connection with the PET substrate, which was far lower than its stretching limit (Fig. S18), ensuring the normal operation of the whole device.

### Wireless charging integrated microsystem for glucose monitoring

**Figure 5.**
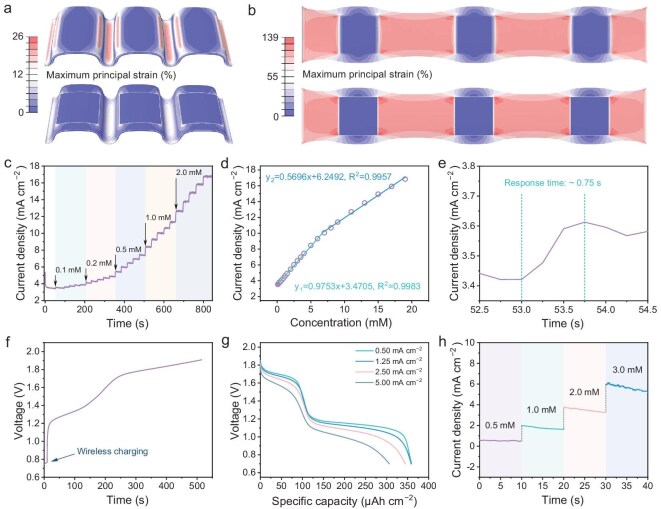
Collaboration and sensing capability of the modular integrated microsystem. (a, b) FEA results of strain distribution for the microsystem in 180° bent state (a) and 50% stretched state (b). The top image shows the entire device; the bottom image removes the top Ecoflex layer. (c) Current response curves of NCO to sequential addition of different glucose concentrations (0.1 mM, 0.2 mM, 0.5 mM, 1.0 mM and 2.0 mM, each added five times). (d) Linear fitting relationship between current density and glucose concentration. (e) Response time of NCO after adding 0.1 mM glucose. (f) Wireless charging curve of ASMBs. (g) Galvanostatic discharge profiles of ASMBs after wireless charging. (h) Current response of NCO to different glucose concentrations under ASMBs power supply.

To validate the application potential at the integrated microsystem level, we chose sweat glucose monitoring as a practical example, given that sweat is a vital physiological fluid containing various biomarkers that can indicate the health status of the human body [46–49]. For this purpose, we fabricated wireless receiving coils, ASMBs, and a glucose sensor by fully 3D-printing, and extended the above ‘island-bridge’ structure design to the microsystem level for integration. To demonstrate the effectiveness of the proposed strategy in the microsystem, strain simulation was also performed. Similarly, the strain is primarily concentrated in the elastic regions connecting the rigid ‘islands’ (Fig. 5a, b and Fig. S19), which ensures mechanical stability at the system level. Besides, it is particularly noteworthy that each module in the microsystem was fabricated by 3D-printing EG-based inks, which greatly reduced the complexity of the fabrication process and enhanced the compatibility of the whole microsystem.

Before evaluating the modular integrated microsystem for glucose monitoring, we first characterized the property of the synthesized NCO (Fig. S20), showing a sea urchin–like morphology (Fig. S21). It is noted that the abundant nanowire structure on the surface could facilitate its contact with analytes, thereby improving sensing performance. Further, the CNTs were introduced and mixed with NCO to accelerate the electron transfer and response rate. Finally, the glucose sensor was obtained after drop-casting the mixture onto the 3D-printed EG working electrode. Sensitivity tests revealed that the NCO material was capable of responding to glucose concentrations as low as 0.1 mM, with an increasing response current as glucose concentration rose (Fig. 5c). By fitting the response current density and concentration, it is disclosed that the NCO displayed different sensitivities in the low concentration (0.1–7.0 mM) and high concentration (7.0–19.0 mM) ranges (Fig. 5d), which may be induced by the adsorption of catalytic oxidation products on the surface of the working electrode at high concentrations, hindering diffusion of the analyte [50]. Moreover, the NCO material delivered an extremely short response time of only 0.75 s (Fig. 5e). Additionally, the NCO also exhibited excellent selectivity (Fig. S22) and reversibility (Fig. S23), ensuring its suitability in glucose monitoring microsystems.

Subsequently, the comprehensive property of the modular integrated microsystem was further evaluated. First, we optimized the parameters of the EG receiving coils (Fig. S24), thereby achieving a wireless charging efficiency of 34.6% (Fig. S25). It can be noted from Fig. 5f that the 3D-printed EG receiving coils reliably charged the ASMBs to the cutoff voltage of 1.9 V. Subsequently, the ASMBs after wireless charging delivered close discharge capacity at different current densities (Fig. 5g), demonstrating that wireless charging offered a comparable effect to galvanostatic charging, and also corroborating the excellent rate performance of ASMBs. Besides, it is validated that the wireless charging speed had no significant effect on the discharge capacity of the ASMBs (Fig. S26), underscoring the fast-charging potential of this wireless charging approach. Further, the ASMBs were utilized as micropower sources to drive the NCO sensor. It was noticed that the NCO sensor powered by ASMBs successfully gave out distinct current responses to glucose with varying concentrations (Fig. 5h), demonstrating its potential for real-time monitoring of sweat glucose. Impressively, the entire microsystem maintained nearly unchanged performance even during dynamic stretching (Fig. S27) and after 1000 stretching cycles (Fig. S28), highlighting the effectiveness of our strategy in construction of energy harvest-storage-application modular integrated microsystems. Furthermore, the infrared thermal image (Fig. S29) manifested that the peak temperature of the microsystem during wireless charging was only 27.8°C (with a background temperature of 20.0°C), verifying the safety of our microsystem for wearable applications. Therefore, our 3D-printed modular integrated microsystem exhibits remarkable compatibility, mechanical robustness and customizability (Table S1), evidencing its immense potential in wearable electronics.

## CONCLUSION

In summary, we developed a highly compatible and stretchable modular integrated microsystem consisting of wireless receiving coils, ASMBs, and glucose monitoring sensors. Attributed to excellent electron/ion transfer pathways in NVTP/EG microelectrode and appropriate matching between electrode and electrolyte, the ASMBs demonstrated a high areal energy density of 1.24 mWh cm^−2^ and favorable cyclability. The wireless receiving coils could effectively charge the ASMBs and sequentially power the sensor to realize rapid and highly sensitive detection to glucose in simulated sweat, demonstrating the applicability of our modular integrated microsystem containing energy harvest-storage-consumption modules. Remarkably, the elaborate structure design enabled the microsystem to maintain stable operation after undergoing repeated stretching cycles of 50% tensile strain, presenting a significant opportunity for wearable electronics. We believe that the proposed 3D-printing strategy and structural design can be extended to the large-scale fabrication of highly compatible and customizable energy-related integrated microsystems toward wearable electronics.

## Supplementary Material

nwaf364_Supplemental_File
