# Supplementary material for: 3D-printed stretchable modular integrated microsystems toward sweat monitoring powered by wireless charging sodium-ion micro-batteries
Source: Natl Sci Rev. 2025 Aug 30;12(10):nwaf364. doi: 10.1093/nsr/nwaf364 (PMC12485988; doi:10.1093/nsr/nwaf364)
Supplement: nwaf364_Supplemental_File [file nwaf364_supplemental_file.pdf]

Supplementary Information for

3D-printed stretchable modular integrated microsystems toward sweat monitoring powered by wireless charging sodium-ion micro-batteries

Zhihao Ren[1,6], Xiaoyu Shi[1,5,*], Endian Yang[1], Lanxiu Ni[2], Zhuobin Guo[1,6], Bin Li[1,6], Yin Wu[1], Yuxin Ma[1,6], Junwei Sun[1], Yangyang Liu[1], Chunsheng Li[2], Jiaxin Ma[1,5], Xiao Wang[1,5], Feng Zhou[1,5], Fangyuan Hu[4], Liang Feng[2], Quan Shi[3] and Zhong-Shuai Wu[1,5,*]

[1]State Key Laboratory of Catalysis, Dalian Institute of Chemical Physics, Chinese Academy of Sciences, Dalian 116023, China;

[2]Department of Instrumentation and Analytical Chemistry, CAS Key Laboratory of Separation Science for Analytical Chemistry, Dalian Institute of Chemical Physics, Chinese Academy of Sciences, Dalian 116023, China;

[3]Thermochemistry Laboratory, Dalian Institute of Chemical Physics, Chinese Academy of Sciences, Dalian 116023, China;

[4]School of Materials Science and Engineering, State Key Laboratory of Fine Chemicals, Dalian University of Technology, Dalian 116024, China;

[5]Dalian National Laboratory for Clean Energy, Chinese Academy of Sciences, Dalian 116023, China;

[6]University of Chinese Academy of Sciences, Beijing 100049, China

*Corresponding authors: Xiaoyu Shi; Zhong-Shuai Wu

E-mails: shixiaoyu@dicp.ac.cn; wuzs@dicp.ac.cn

Materials and Methods

Synthesis of EG nanosheets, NVTP@C and NCO

EG was synthesized using the electrochemical exfoliation reported previously [1]. In a 1 M (mol L^{-1}) $NaBF_4$ solution (AR, J&K Scientific Co., Ltd.), commercial graphite paper was used as the negative electrode, and a platinum sheet was used as the positive electrode, with a direct current (DC) bias voltage of +10 V applied for electrochemical exfoliation. The resulting expanded graphite was washed multiple times with deionized (DI) water and ethanol and then redispersed in ethanol. Finally, the dispersion was ultrasonicated for 2 h to obtain an EG nanosheet suspension (~3 g L^{-1}).

NVTP@C was synthesized using the sol-gel method reported previously [2]. First, 0.01 mol Na_2CO_3 (AR, Sinopharm Co., Ltd.), 0.01 mol NH_4VO_3 (AR, Aladdin Co., Ltd.), 0.03 mol $NH_4H_2PO_4$ (AR, Aladdin Co., Ltd.), and 0.01 mol citric acid monohydrate (AR, Sinopharm Co., Ltd.) were dissolved in 100 mL of DI water. Separately, 0.01 mol $(CH_3CH_3CH_3CO)_4Ti$ (AR, MERYER Co., Ltd.) was dissolved in 50 mL of ethanol. These two solutions were then mixed and stirred in a water bath at 80 ℃ for 12 h to obtain the precursor. Under an Ar atmosphere, the precursor was first held at 350 ℃ for 5 h, then heated to 800 ℃ at a rate of 5 ℃ min^{-1}, and calcined for 12 h to produce NVTP@C.

NCO was synthesized using the hydrothermal method reported previously [3]. First, 4 mmol $NiCl_2 \cdot 6H_2O$ (AR, Macklin Co., Ltd.), 8 mmol $CoCl_2 \cdot 6H_2O$ (AR, Sinopharm Co., Ltd.), and 16 mmol urea (AR, Aladdin Co., Ltd.) were dissolved uniformly in 60 mL of DI water. The resulting solution was transferred to a 100 mL polytetrafluoroethylene-lined autoclave and maintained at 120 ℃ for 6 h. After cooling to room temperature, the product was washed several times with DI water and ethanol by centrifugation. Finally, the dried sample was calcined in air at 300 ℃ for 3 h to obtain sea urchin-like NCO.

Preparation of EG-ink and NVTP/EG-ink

The binder-free 3D-printing ink was prepared using a grinding method. First, the ethanol dispersion of EG was vacuum filtered to obtain solid EG nanosheets. For EG-ink, the EG was thoroughly mixed with an appropriate amount of glycerol and ground to achieve well-dispersed EG-ink. For NVTP/EG-ink, NVTP and EG were mixed at a mass ratio of 7:3, followed by the addition of an appropriate amount of glycerol and sufficient grinding to obtain well-dispersed NVTP/EG-ink. Increasing the NVTP content may lead to electrode cracking, probably because the EG content is too low to form a complete conductive pathway.

Preparation of NaTFA aqueous electrolytes

To prepare 1 m (mol kg^{-1}), 5 m, 10 m, 20 m, 25 m, and 30 m NaTFA electrolytes, 1 mmol, 5 mmol, 10 mmol, 20 mmol, 25 mmol, and 30 mmol NaTFA (AR, ChemXYZ Co., Ltd.) were dissolved in 1 g DI water, respectively. For the 3D-printing gel electrolyte, 0.15 g of PVA (molecular weight ~205,000, Macklin Co., Ltd.) was added with stirring continuously at 90 ℃ for 2 h.

Fabrication of wireless coils, ASMBs, glucose sensors and stretchable modular integrated microsystems

3D-printing was performed by virtue of a 3D dispensing machine (ZZ-221, ZhongZhi Automation Co., Ltd.). First, the prepared EG-ink, NVTP/EG-ink, Ag/AgCl-ink (Julong Technology Co., Ltd.), and gel electrolyte were loaded into the syringes. Then, coil patterns, interdigitated microelectrodes, and sensing electrodes were printed on a PET substrate according to computer-designed micropatterns. The nozzle inner diameters for EG-ink, NVTP/EG-ink, Ag/AgCl-ink, and gel electrolyte were 420 μm, 420 μm, 160 μm, and 160 μm, respectively. The printing pressure and speed were approximately 70 psi and 1 mm s^{-1} for EG and NVTP/EG inks, and 20 psi and 3 mm s^{-1} for Ag/AgCl-ink and gel electrolyte. The printed micropatterns were then placed in a vacuum drying oven and dried at 75 ℃ for 12 h, followed by 110 ℃ for 12 h.

The obtained receiving coils can be used directly, while the ASMBs are

encapsulated for use after 3D-printing gel electrolyte on the electrodes. For the sensors, the following steps were performed. 0.05 g of chitosan (AR, Macklin Co., Ltd.) and 0.1 g of acetic acid (AR, Xilong Chemical Co., Ltd.) were dissolved in 5 mL of DI water. Separately, 0.025 g of NCO and 0.01 g of CNTs were added to 2.5 mL of phosphate buffer solution (pH = 7.2, Macklin Co., Ltd.). Subsequently, the above two solutions were mixed thoroughly, and 20 μL was drop-cast onto the EG working electrode and dried for use.

For the stretchable modular integrated microsystem, a three-layer structure was designed. First, the A and B components of Ecoflex 00-30 (Smooth-On Co., Ltd.) were thoroughly mixed at a 1:1 mass ratio, degassed to remove bubbles, and cast onto a flat substrate. The mixture was cured at 50 ℃ for 15 minutes. Next, the previously prepared wireless charging module, ASMBs module, and glucose sensing module were placed on the surface of the cured Ecoflex layer. GaInSn liquid metal was used as the conductive wires to connect the modules. A small piece of plastic film was placed over the interdigitated electrode section of the ASMBs to prevent electrolyte contamination. Finally, another layer of Ecoflex was cast over the assembly and cured at 50 ℃, resulting in the stretchable modular integrated microsystem.

Materials characterization

The morphology and structure of EG, NVTP@C and NCO were characterized by XRD (SmartLab), Raman spectroscopy (NanoWizard), AFM (NanoWizard), TEM (F30) and SEM (Quanta 200F and JSM-7900F) techniques. The structure of EG and NVTP/EG electrodes were characterized by SEM (Quanta 200F) and step profiler (Alpha step D-600). The rheological properties of EG-ink, NVTP/EG-ink and gel electrolyte were characterized by a rheometer (Anton Paar MCR 302). The properties of 1 m and 30 m NaTFA electrolytes were examined by FTIR spectroscopy (Nicolet iS50) and Raman spectroscopy (NanoWizard). The tensile test of Ecoflex was performed by a tensile testing machine (DR-5010AS).

Electrochemical and mechanical measurement

For ASMBs, the devices were packaged in an acrylic plate and linked to an electrochemical workstation (CHI760E) or a battery tester (LAND) by commercially available conductive silver paste and Cu conductive tape. The electrochemical impedance spectroscopy (EIS) was conducted in the frequency range from 0.01 Hz to 100 kHz with an amplitude of 5 mV. The performance of NVTP||Na half cells were tested in 2016-type coin cells with polypropylene films (Celgard 2500) as the separator and 1 M $NaPF_6$ in DIGLYME (Dodochem Co., Ltd.) as the electrolyte. The electrochemical data for ASMBs and coin cells were collected after pre-cycling several cycles at 28 ℃. The electrochemical stability window of 1–30 m NaTFA electrolytes were evaluated by LSV using glassy carbon as working electrode, Pt as counter electrode and Ag/AgCl as reference electrode, respectively. For glucose sensors, the *i-t* and CV curves were obtained by progressively adding glucose to 0.15 M NaOH solution at a bias pressure of +0.7 V in a three-electrode system using EG supported NCO/CNTs as working electrode, Pt as counter electrode and Hg/HgO as reference electrode, respectively. NaOH was used because non-enzymatic active materials commonly function under strongly alkaline conditions (pH ≥ 13), which promote glucose dehydrogenation and reduce the oxidation overpotential, thereby minimizing interference from other analytes [4]. For microsystem, the performance of wireless charging was tested with a homemade wireless charging platform (Fig. S30), where the input voltage was controlled through a DC power supply (Victor Tech Co., Ltd.). The glucose sensors were tested in a two-electrode system with EG supported NCO/CNTs electrode and Ag/AgCl electrode under ASMBs power supply. The infrared images of the device were recorded by an infrared imager (Fluke Co., Ltd.). Mechanical properties of the devices were carried out with a custom digital stepper.

Simulation

Molecular dynamics simulations were performed using the COMPASS III force field within the Forcite module of Materials Studio software. A time step of 1 fs was employed. Electrolyte systems were equilibrated in the isothermal-isobaric ensemble using the Berendsen barostat with a decay constant of 0.1 ps for 1 ns, maintaining a

temperature of 298.15 K controlled by a Nosé thermostat. Subsequently, a production run of 1 ns was carried out in the canonical ensemble at temperatures of 298.15 K. The simulation duration was sufficient to ensure that the electrolyte systems reached the equilibrium.

Finite element simulation analysis of the "island-bridge" structure was carried out using Abaqus software. In the designed geometric model, PET was considered as an elastic material (Young's modulus of 3700 MPa, Poisson's ratio of 0.3), while Ecoflex was considered as a hyperelastic material (stress-strain data from Fig. S18, Poisson's ratio of 0.5). For PET, 4-node quadrilateral shell elements with reduced integration (S4R) were used, and 3D 8-node linear brick elements with reduced integration and hybrid formulation (C3D8RH) were employed for Ecoflex. The constraints between PET and Ecoflex, as well as between the two layers of Ecoflex, were defined as tie constraints. Liquid metal exhibits fluid-like behavior, so it was not included in the simulated model. Both the tensile and bending tests were conducted by fixing one end and moving the other.

Supplementary Figures

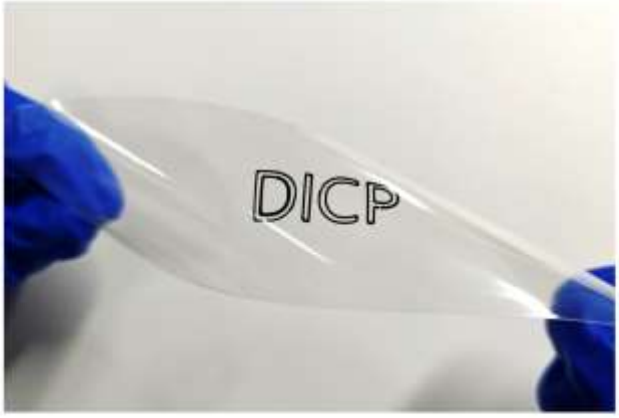

Fig. S1 Photograph of the 3D-printed “DICP” patterns in a bent state.

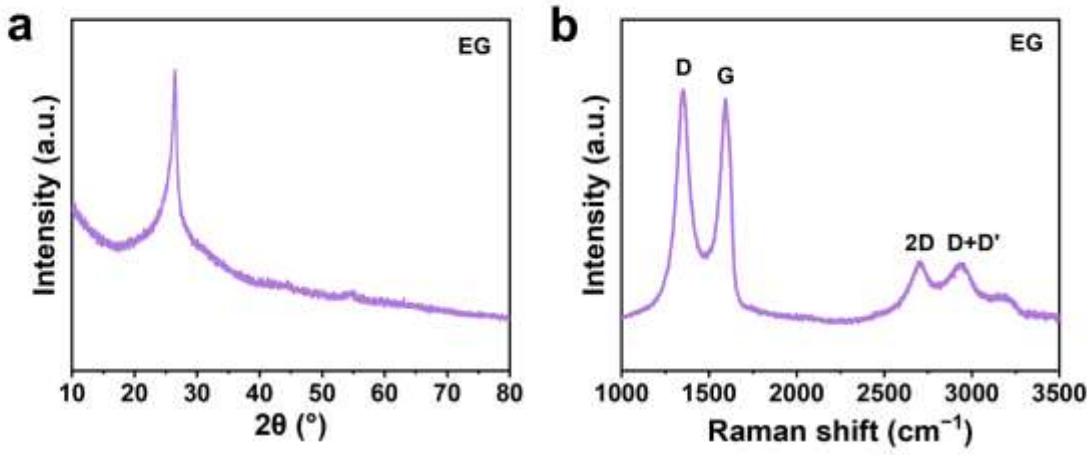

Fig. S2 (a) XRD pattern and (b) Raman spectrum of EG.

Supplementary Data Note: The peaks presented in the Raman spectra are indicative of defective graphene, likely due to the introduction of fluorinated functional groups during the exfoliation process [1].

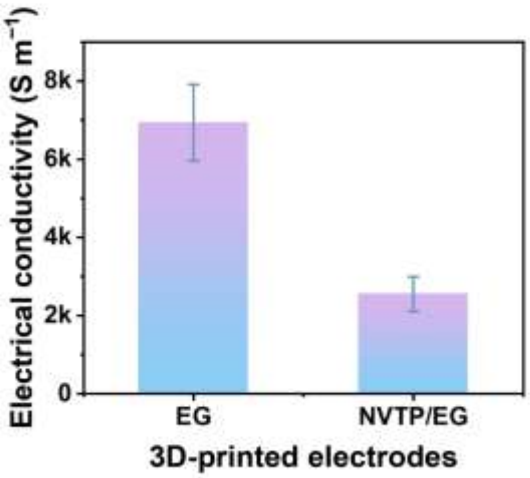

Fig. S3 Electrical conductivity of the 3D-printed EG and NVTP/EG electrodes.

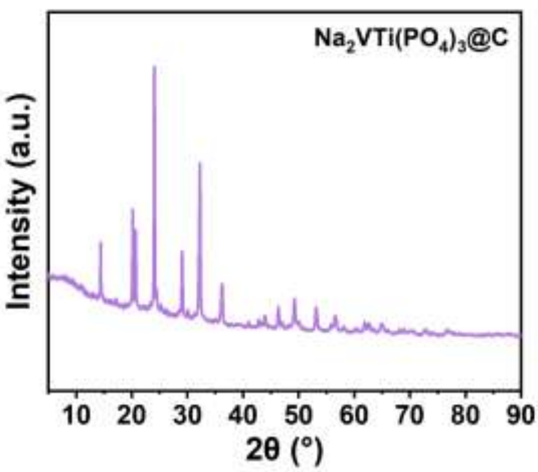

Fig. S4 XRD pattern of the NVTP@C material.

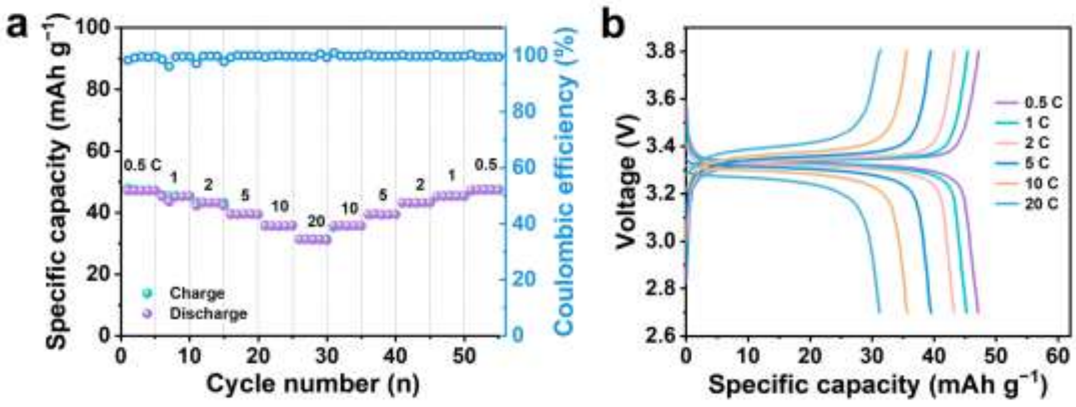

Fig. S5 Electrochemical performance of NVTP@C obtained at 2.7–3.8 V (*vs.* Na^+/Na). (a) Rate performance. (b) Galvanostatic charge-discharge profiles of NVTP@C tested at different rates. (1 C = 62 mAh g^{-1}).

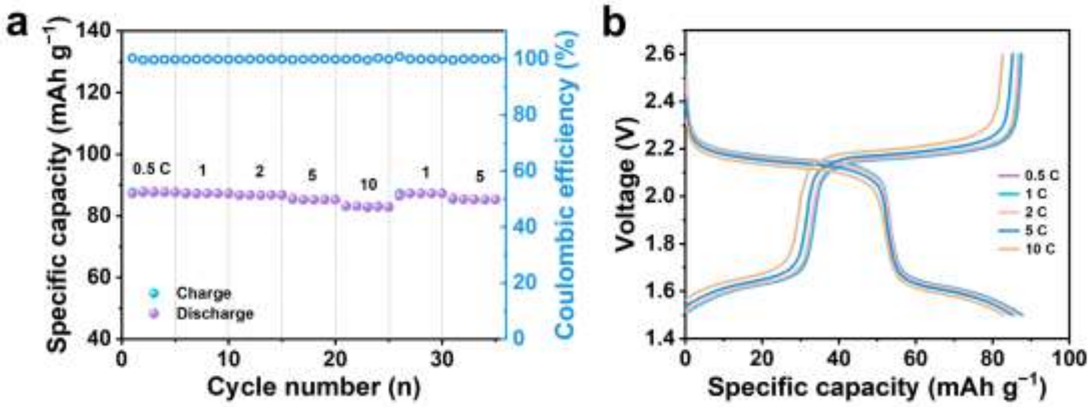

Fig. S6 Electrochemical performance of NVTP@C obtained at 1.5–2.6 V (*vs.* Na^+/Na). (a) Rate performance. (b) Galvanostatic charge-discharge profiles of NVTP@C tested at different rates. (1 C = 128 mAh g^{-1}).

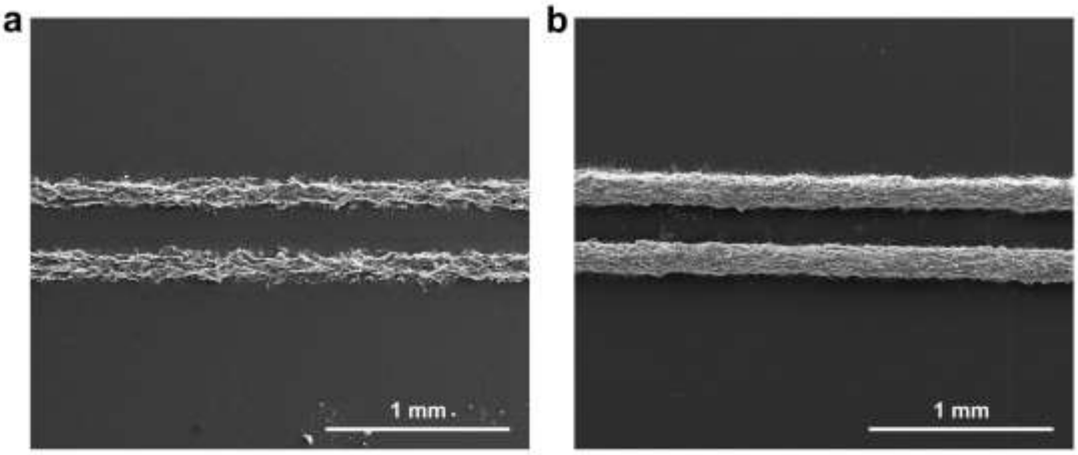

Fig. S7 3D-printing resolution of the (a) EG and (b) NVTP/EG electrodes.

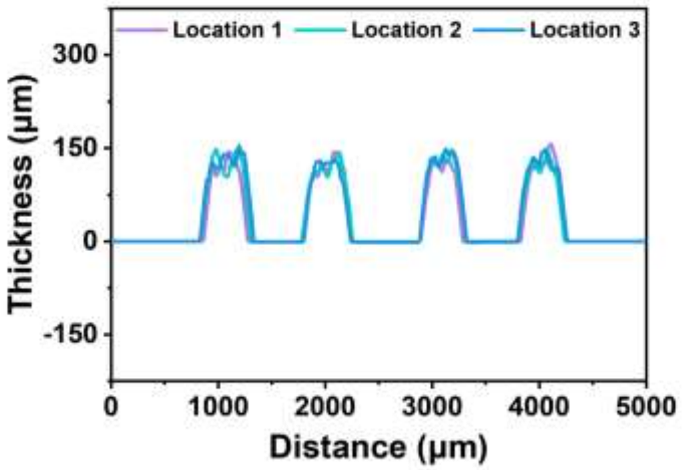

Fig. S8 Height profiles of the 3D-printed ASMBs electrodes at randomly selected locations.

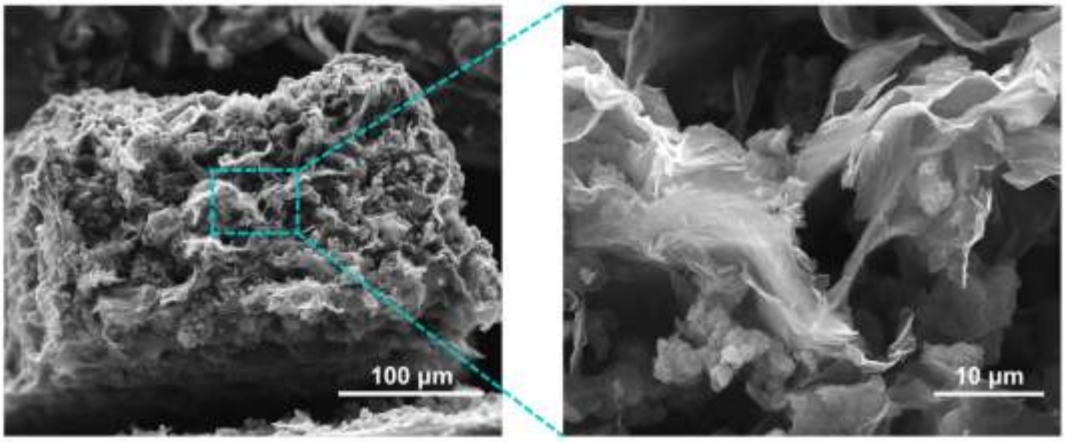

Fig. S9 Cross-section SEM images of the NVTP/EG electrode at different magnification.

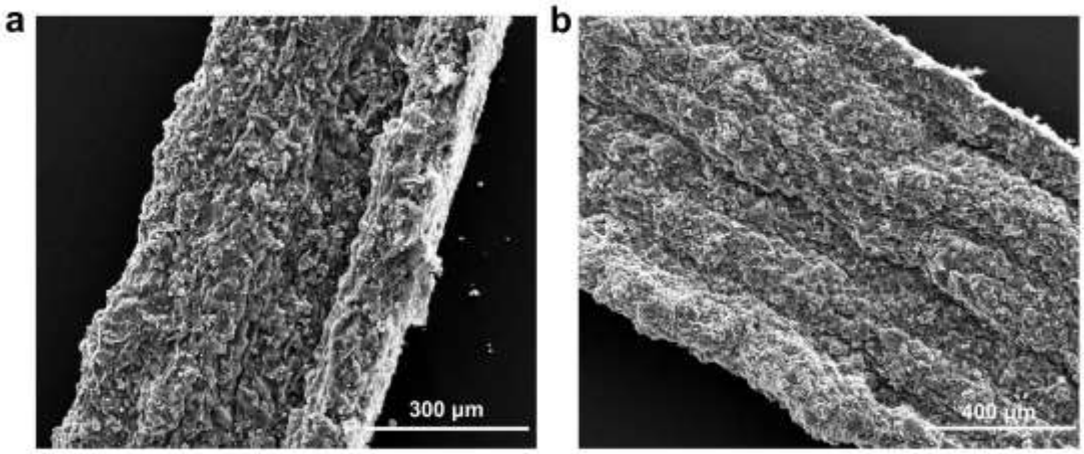

Fig. S10 Side-view SEM images of the 3D-printed NVTP/EG electrodes with (a) 4 layers and (b) 6 layers.

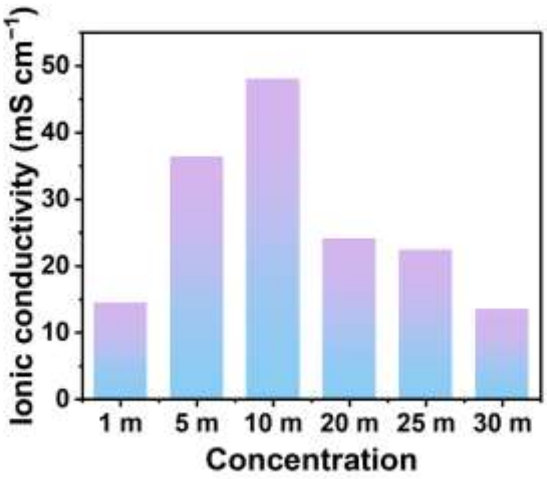

Fig. S11 Ionic conductivity of NaTFA electrolytes at different concentrations.

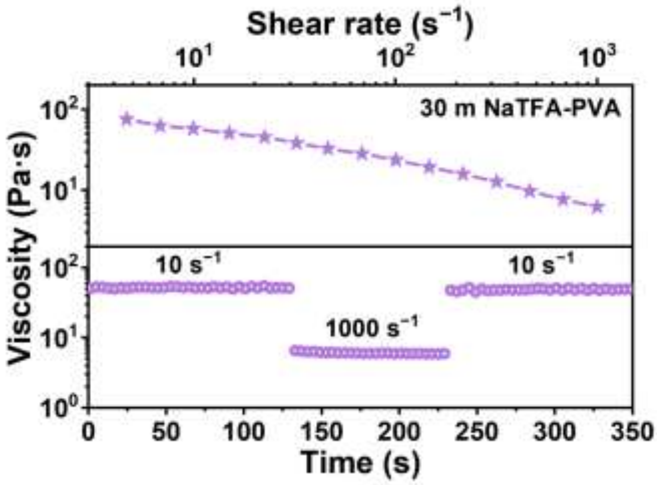

Fig. S12 Rheological behavior of 30 m NaTFA-PVA gel electrolyte.

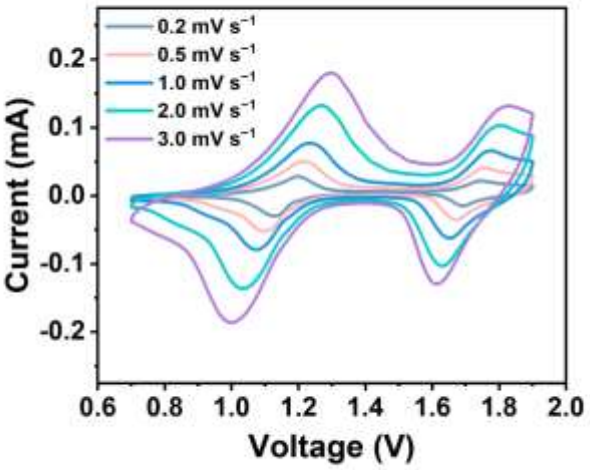

Fig. S13 CV curves of the ASMBs-2L^{+}1L^{-}.

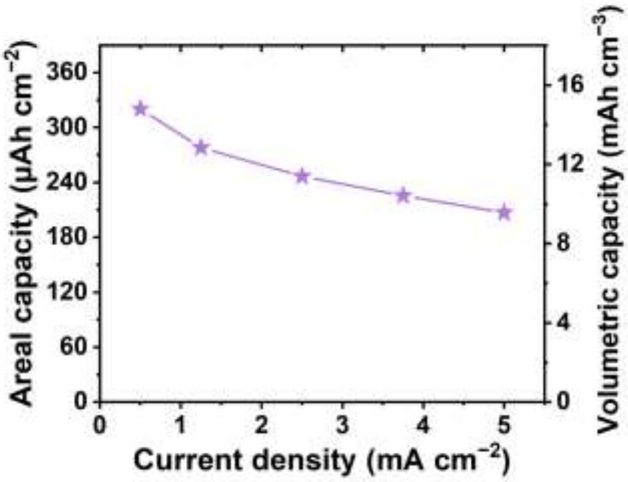

Fig. S14 Areal capacity and volumetric capacity of ASMBs-2L^{+}1L^{-} as a function of current density.

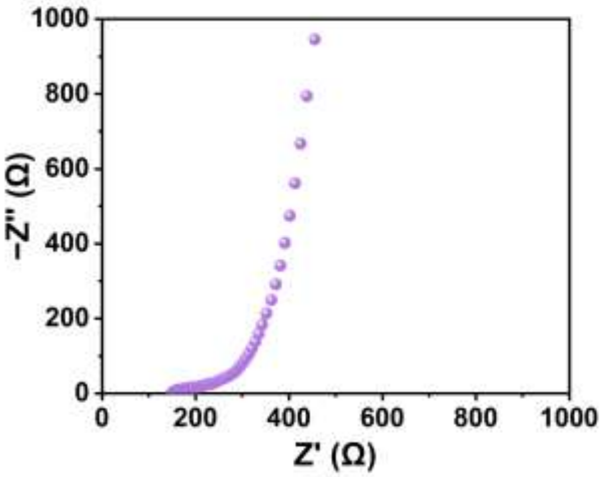

Fig. S15 EIS of the ASMBs-2L^{+}1L^{-}.

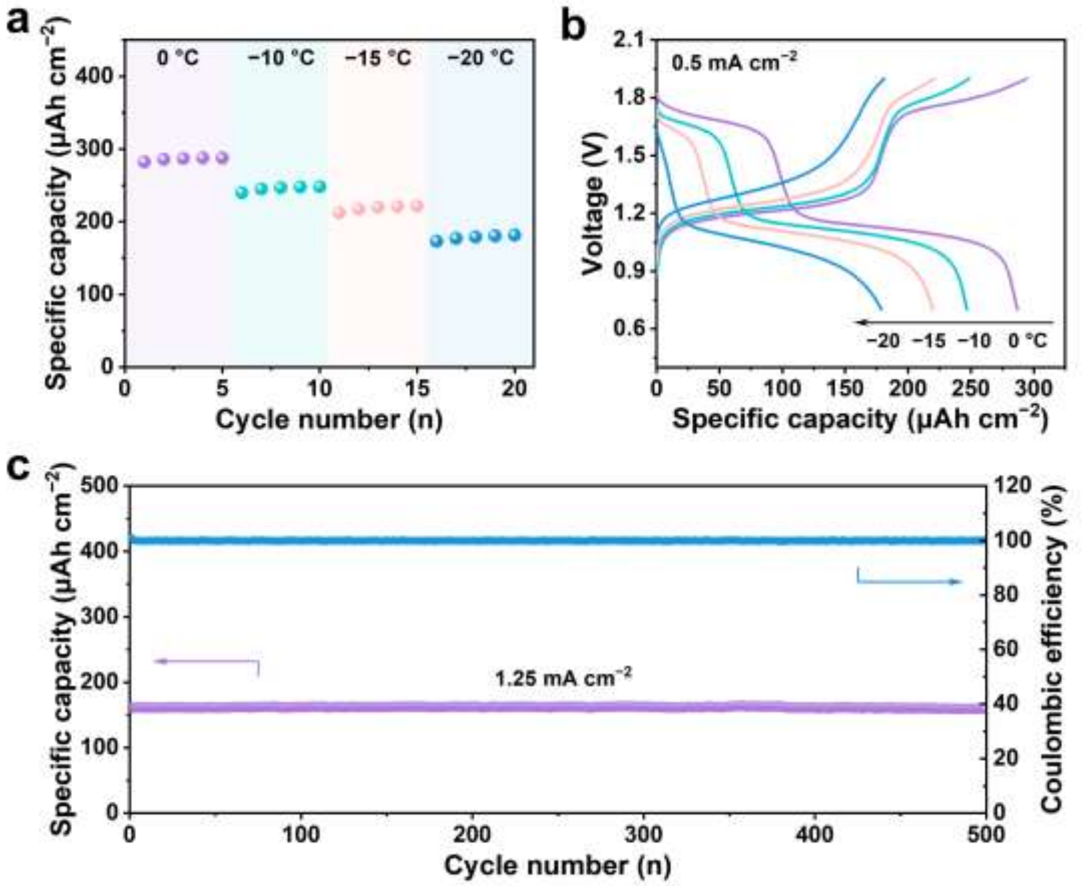

Fig. S16 (a) Specific capacity comparison and (b) specific capacity-voltage curves of ASMBs-2L^{+}1L^{-} at low temperature. (c) Cyclic stability of ASMBs-2L^{+}1L^{-} at −15 °C.

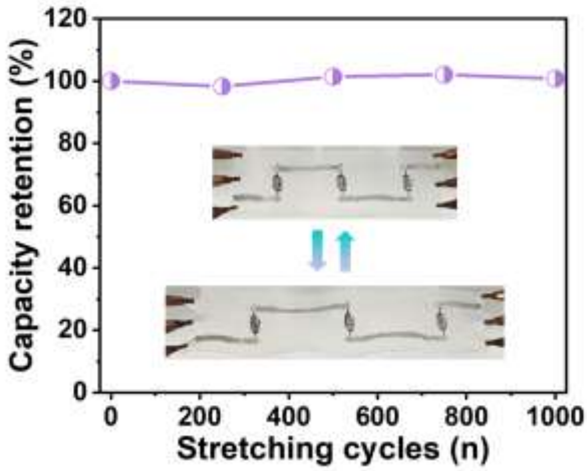

Fig. S17 Capacity retention of stretchable ASMB arrays connected in series after different cycles of 0–50% stretching. The insets are corresponding photographs.

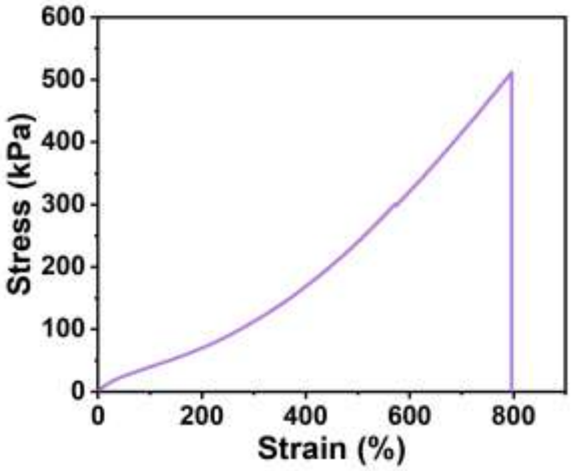

Fig. S18 Stress-strain curve of the Ecoflex elastomer.

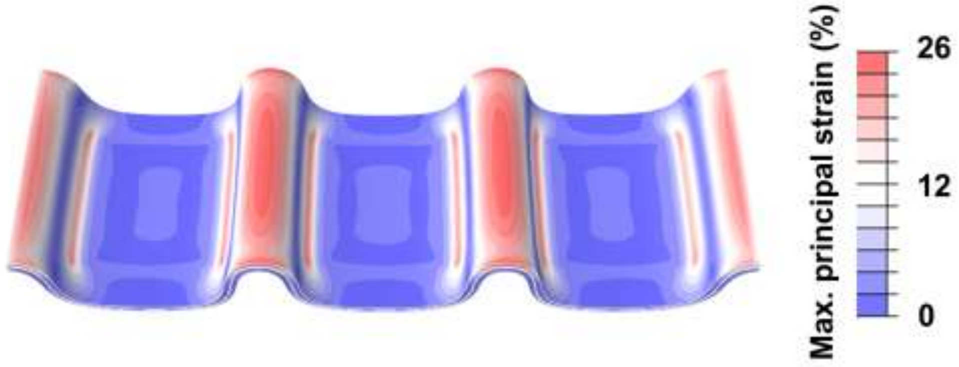

Fig. S19 FEA results of strain distribution for the modular integrated microsystem (bottom) in 180 °bent state.

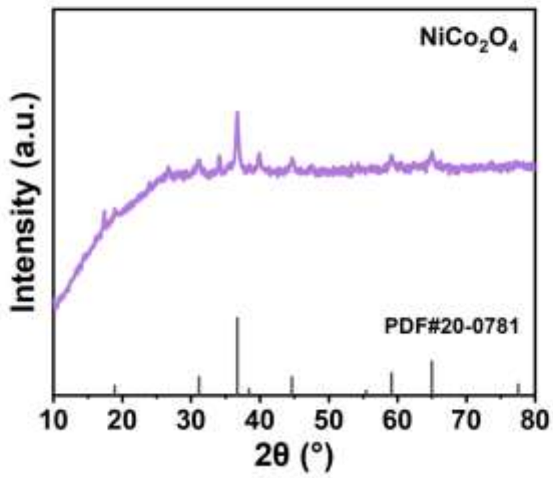

Fig. S20 XRD pattern of the NCO sensing material.

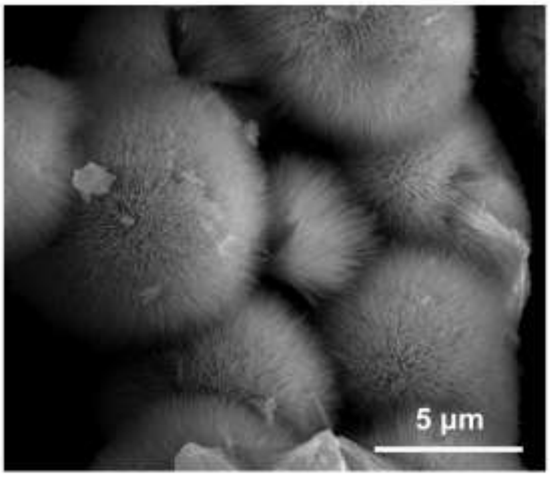

Fig. S21 SEM image of the NCO sensing material.

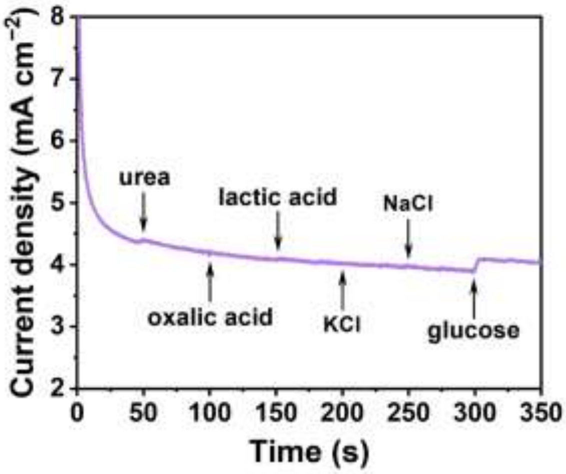

Fig. S22 Current response of NCO to different analytes (0.25 mM urea, 0.25 mM oxalic acid, 0.25 mM lactic acid, 0.25 mM KCl, 0.25 mM NaCl, and 0.25 mM glucose).

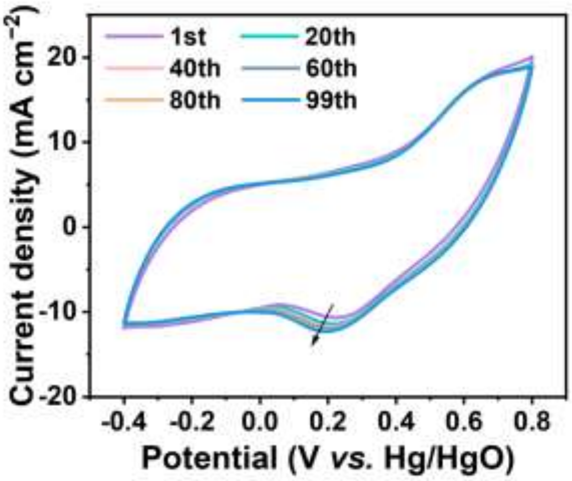

Fig. S23 CV curves of NCO in 0.5 mM glucose solution.

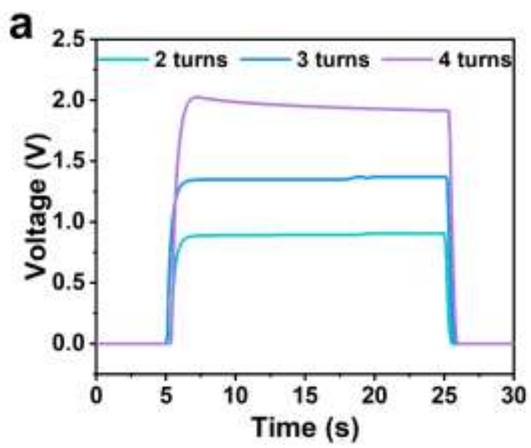

Fig. S24 Voltage signals induced in EG receiving coils with different (a) turn number and (b) area.

Supplementary Data Note: The induced voltage on the coils increased with a higher number of turns and reduced area, consistent with the principle of electromagnetic induction, where the induced electromotive force is proportional to the rate of change of magnetic flux. Taking into account the voltage of ASMBs and the size of microsystem, the final EG coils were designed with 4 turns and an area of 4 cm^2.

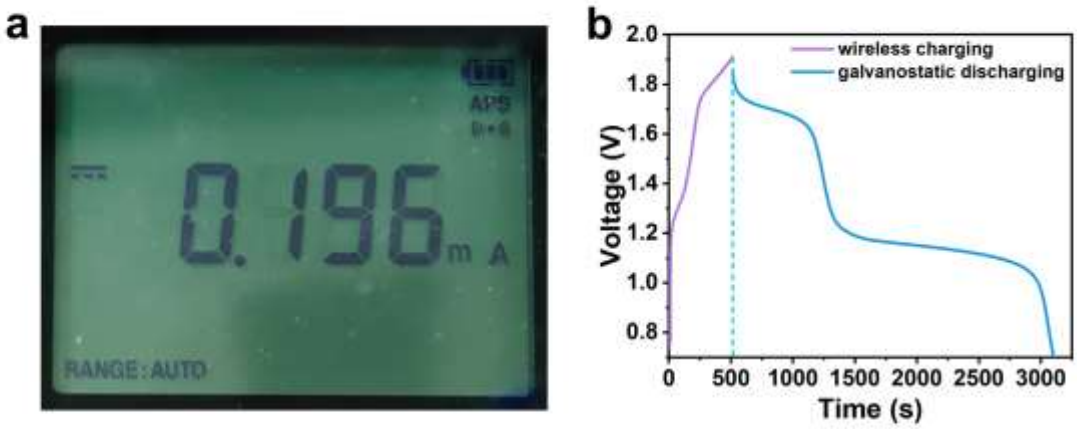

Fig. S25 (a) Current value induced in EG receiving coils. (b) Wireless charging and corresponding galvanostatic discharging (0.02 mA) curves of ASMBs-$2L^{+}1L^{-}$.

Supplementary Data Note: The wireless charging efficiency from the EG receiving coils to the ASMBs was estimated based on the following equation:

$$\eta = \frac{E_{ASMB}}{E_{Coil}} \times 100\% = \frac{\int U(t)_{ASMB} dt \cdot I_{ASMB}}{U_{Coil} \cdot I_{Coil} \cdot t_{Coil}} \times 100\%$$

where E_{ASMB} was the discharge energy of ASMBs, E_{Coil} was the output energy of EG coils, $U(t)_{ASMB}$ was the voltage of the ASMBs that varied with discharge time, I_{ASMB} was the discharge current (0.02 mA) of ASMBs, U_{Coil} was the induced voltage of EG coils (1.95 V, Fig. S24), I_{Coil} was the induced current (0.196 mA, Fig. S25a) of EG coils, t_{Coil} was the output time (501 s, Fig. S25b) of EG coils. According to the calculation, E_{ASMB} was 18.4 μWh and E_{Coil} was 53.2 μWh. Therefore, the wireless charging efficiency was 34.6%.

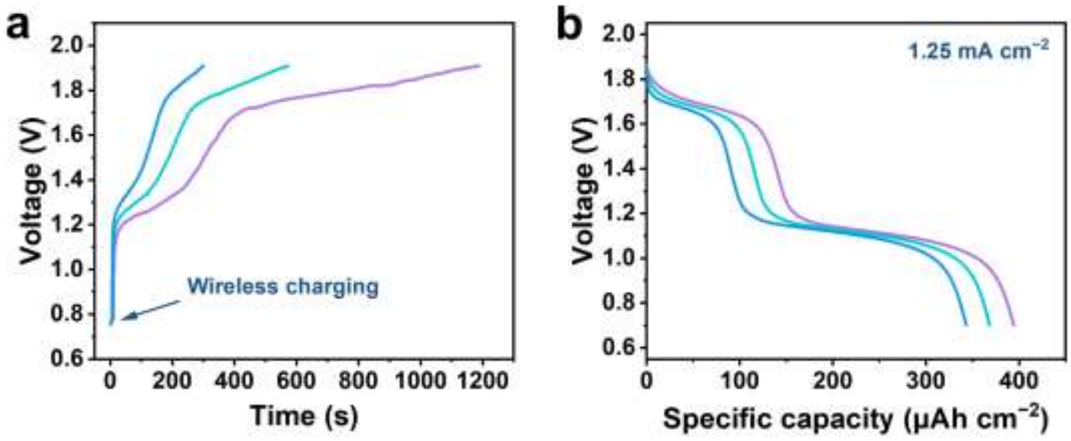

Fig. S26 (a) Wireless charging curves at different charging rates and (b) corresponding galvanostatic discharging profiles of ASMBs-2L$^+$1L$^-$.

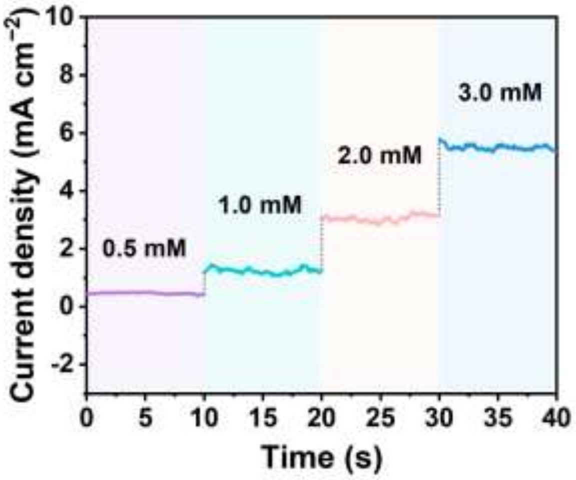

Fig. S27 Current response plots of NCO in microsystem under a dynamic stretching rate of 5% s^{-1}.

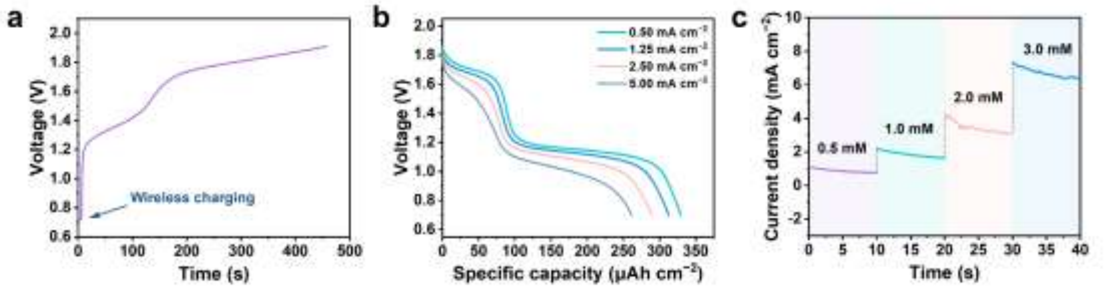

Fig. S28 (a) Wireless charging curve, (b) galvanostatic discharge profiles of ASMBs and (c) current response plots of NCO in microsystem after 1000 stretching cycles from 0% to 50% strain.

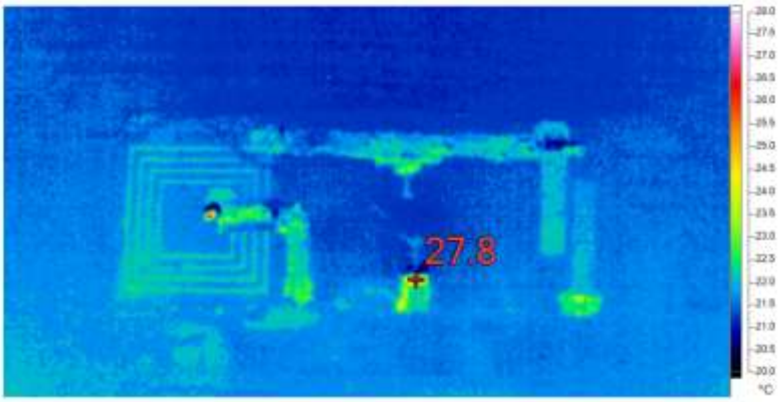

Fig. S29 Infrared thermal image of the modular integrated microsystem during the wireless charging process.

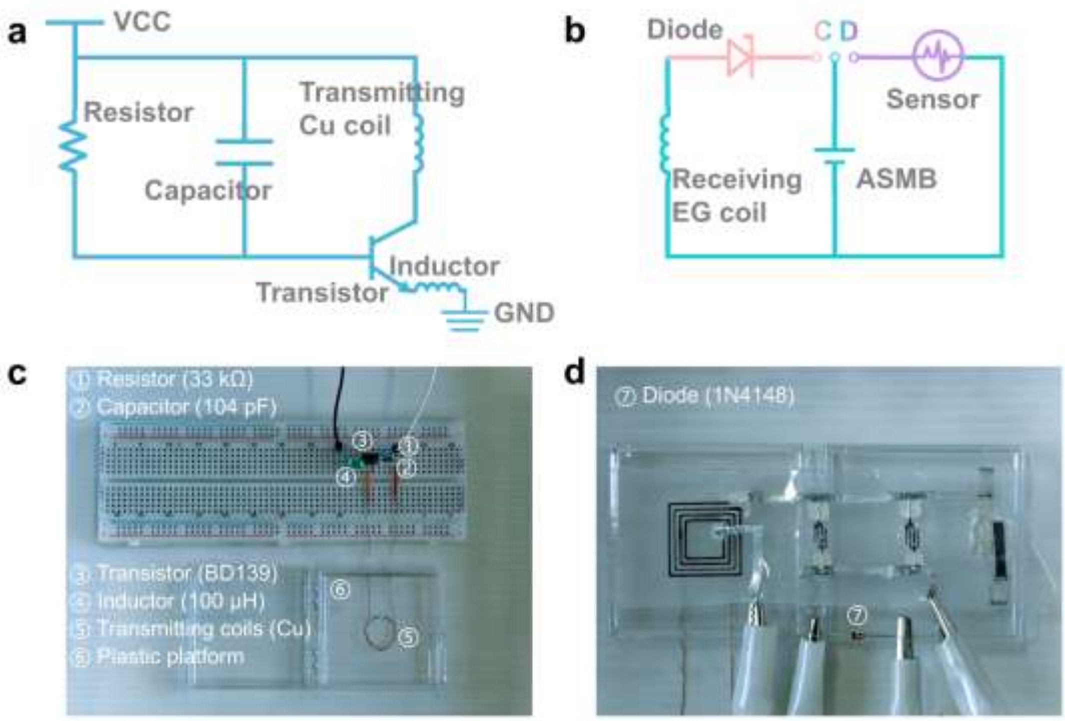

Fig. S30 Schematic and optical images of the wireless charging platform. (a, b) Circuit diagram of the transmitting port (a) and receiving port (b). (c, d) Optical images of the transmitting port (c) and receiving port (d).

Supplementary Data Note: In general, the wireless charging platform consists of a transmitting port (Fig. S30a, c) and a receiving port (Fig. S30b, d). The transmitting converts input DC to high-frequency alternating current (AC) via an oscillator circuit, driving Cu transmitting coils to generate a rapidly changing magnetic field based on electromagnetic induction. This magnetic field induces an alternating electromotive force in the EG receiving coils placed above the Cu transmitting coils, producing AC. To utilize this wirelessly harvested AC for charging, the EG receiving coils are connected to rectifying diodes (point C in Fig. S30b). These diodes rectify the AC into DC, ensuring that charges accumulate on the positive and negative electrodes of the ASMBs. When connected at point D (Fig. S30b), the stored energy in the ASMBs is released to power the sensor. Thus, the wireless transfer occurs specifically between the transmitting and receiving coils. Wire connection is necessary for the initial power input and the final connection to the ASMBs.

Table S1 Performance comparison of our modular integrated microsystem with other reported microsystems.

Preparation technologies	Energy harvesting devices	Energy storage devices (Energy density)	Stretchability	Retention rate (Stretching cycles)	Energy application devices	Connection method	References
lithography + electrodeposition	-	micro-supercapacitors (3.2 mWh cm^{-3})	40% (biaxial)	95% (1000)	NO_2 gas sensor	Au	[5]
3D printing	wireless coils	micro-supercapacitors (~0.164 mWh cm^{-2})	30% (biaxial)	-	light emitting diodes	Cr/Au	[6]
laser etching + spraying	triboelectric nanogenerators	micro-supercapacitors	100% (uniaxial)	-	strain sensor	external wiring	[7]
chemical vapor deposition + inkjet printing	wireless coils + triboelectric nanogenerators	micro-supercapacitors (~0.003 mWh cm^{-2})	30% (uniaxial)	-	light emitting diodes	external wiring	[8]
pre-stretching + mask-assisted printing	wireless coils	Zn-MnO_2 micro-batteries (2.3 mWh cm^{-2})	150% (uniaxial)	~97% (200)	pressure/temperature sensors	Ag foil	[9]
3D printing	wireless coils	NVTP/EG ASMBs (1.24 mWh cm^{-2}/ 15.8 mWh cm^{-3})	50% (uniaxial)	~100% (1000)	glucose sensor	liquid metal	**This work**

Supplementary References

1. Zhou F, Huang H, Xiao C *et al.* Electrochemically scalable production of fluorine-modified graphene for flexible and high-energy ionogel-based microsupercapacitors. *J Am Chem Soc* 2018; **140**: 8198–205.
2. Wang D, Bie X, Fu Q *et al.* Sodium vanadium titanium phosphate electrode for symmetric sodium-ion batteries with high power and long lifespan. *Nat Commun* 2017; **8**: 15888.
3. Wang Q, Wang X, Liu B *et al.* $NiCo_2O_4$ nanowire arrays supported on Ni foam for high-performance flexible all-solid-state supercapacitors. *J Mater Chem A* 2013; **1**: 2468–73.
4. He C, Asif M, Liu Q *et al.* Noble metal construction for electrochemical nonenzymatic glucose detection. *Adv Mater Technol* 2023; **8**: 2200272.
5. Yun J, Lim Y, Jang GN *et al.* Stretchable patterned graphene gas sensor driven by integrated micro-supercapacitor array. *Nano Energy* 2016; **19**: 401–14.
6. Park J, Ahn DB, Kim J *et al.* Printing of wirelessly rechargeable solid-state supercapacitors for soft, smart contact lenses with continuous operations. *Sci Adv* 2019; **5**: eaay0764.
7. Zhang C, Peng Z, Huang C *et al.* High-energy all-in-one stretchable micro-supercapacitor arrays based on 3D laser-induced graphene foams decorated with mesoporous ZnP nanosheets for self-powered stretchable systems. *Nano Energy* 2021; **81**: 105609.
8. Hong S, Lee J, Do K *et al.* Stretchable electrode based on laterally combed carbon nanotubes for wearable energy harvesting and storage devices. *Adv Funct Mater* 2017; **27**: 1704353.
9. Wang Y, Zhao Y, Yu L *et al.* Deformation-tolerant, wireless-charging microbatteries for seamlessly integrated omnidirectional stretchable electronics. *Sci Adv* 2025; **11**: eads6892.
